# Cross-talks between gut microbiota and tobacco smoking: a two-sample Mendelian randomization study

**DOI:** 10.1186/s12916-023-02863-1

**Published:** 2023-04-28

**Authors:** Jiayao Fan, Yuan Zhou, Ran Meng, Jinsong Tang, Jiahao Zhu, Melinda C. Aldrich, Nancy J. Cox, Yimin Zhu, Yingjun Li, Dan Zhou

**Affiliations:** 1 School of Public Health and the Second Affiliated Hospital, Zhejiang University School of Medicine, 388 Yuhangtang Road, Hangzhou, 310058 China; 2 Department of Epidemiology and Health Statistics, School of Public Health, Hangzhou Medical College, 481 Binwen Road, Hangzhou, 310053 China; 3 Department of Biostatistics and Center for Quantitative Sciences, Vanderbilt University Medical Center, Nashville, TN USA; 4 Department of Psychiatry, Sir Run-Run Shaw Hospital, School of Medicine, Zhejiang University, Hangzhou, China; 5 Division of Genetic Medicine, Department of Medicine, Vanderbilt University Medical Center, Nashville, TN USA; 6 Vanderbilt Genetics Institute, Vanderbilt University Medical Center, Nashville, TN USA; 7 Department of Epidemiology & Biostatistics, School of Public Health, Zhejiang University, 388 Yuhangtang Road, Hangzhou, 310058 Zhejiang China; 8 The Key Laboratory of Intelligent Preventive Medicine of Zhejiang Province, Hangzhou, Zhejiang China

**Keywords:** Gut microbiota, Tobacco smoking, Microbiota-gut-brain axis, Metabolite, Mendelian randomization

## Abstract

**Background:**

Considerable evidence has been reported that tobacco use could cause alterations in gut microbiota composition. The microbiota-gut–brain axis also in turn hinted at a possible contribution of the gut microbiota to smoking. However, population-level studies with a higher evidence level for causality are lacking.

**Methods:**

This study utilized the summary-level data of respective genome-wide association study (GWAS) for 211 gut microbial taxa and five smoking phenotypes to reveal the causal association between the gut microbiota and tobacco smoking. Two-sample bidirectional Mendelian randomization (MR) design was deployed and comprehensively sensitive analyses were followed to validate the robustness of results. We further performed multivariable MR to evaluate the effect of neurotransmitter-associated metabolites on observed associations.

**Results:**

Our univariable MR results confirmed the effects of smoking on three taxa (*Intestinimonas*, *Catenibacterium*, and *Ruminococcaceae*, observed from previous studies) with boosted evidence level and identified another 13 taxa which may be causally affected by tobacco smoking. As for the other direction, we revealed that smoking behaviors could be potential consequence of specific taxa abundance. Combining with existing observational evidence, we provided novel insights regarding a positive feedback loop of smoking through *Actinobacteria* and indicated a potential mechanism for the link between parental smoking and early smoking initiation of their children driven by *Bifidobacterium*. The multivariable MR results suggested that neurotransmitter-associated metabolites (tryptophan and tyrosine, also supported by previous studies) probably played a role in the action pathway from the gut microbiota to smoking, especially for *Actinobacteria* and *Peptococcus*.

**Conclusions:**

In summary, the current study suggested the role of the specific gut microbes on the risk for cigarette smoking (likely involving alterations in metabolites) and in turn smoking on specific gut microbes. Our findings highlighted the hazards of tobacco use for gut flora dysbiosis and shed light on the potential role of specific gut microbiota for smoking behaviors.

**Supplementary Information:**

The online version contains supplementary material available at 10.1186/s12916-023-02863-1.

## Background

Cigarette smoking, a major public health threat across the world, causes more than 8 million deaths globally each year. Despite higher awareness of cigarettes’ adverse effects and ongoing efforts on tobacco control, there still exist 22.3% of the global population being regular smokers (made up of 36.7% of the world’s men and 7.8% of all women) [1]. Smoking is highly inherited with an estimated heritability of 44% (66% for males and 21% for females, respectively) [2, 3] and meanwhile is influenced by postnatal environmental conditions (e.g., socioeconomic position, culture). Understanding the modifiable risk factors of smoking as well as its full spectrum of consequence is always an essential and challenging question, especially at the microscale and molecular levels. The term “gut microbiota” refers to all microorganisms that inhabit the human gastrointestinal tract, whose volume reaches trillion level [4]. Due to its intricate and reciprocal symbiotic relationship with the host, the gut microbiota is closely related to human health, not just intestinal diseases [5–7]. The diversity and quantity of intestinal microbiome are in a dynamic balance, which might be disturbed by various factors, such as genetics, aging, living habits, as well as environmental factors (e.g., cigarette smoke exposure) [8, 9]. Indeed, mounting observational evidence has reported that tobacco use was associated with alterations in the gut microbiota composition [10–12]. Taking *Bifidobacterium* (the representative bacteria of probiotics) for example, existing population studies unanimously found that the abundance of *Bifidobacterium* was significantly decreased in current smokers compared with non-smokers, regardless of the ethnicities [13–17]. Instead, smoking cessation, even for short periods, could somewhat restore *Bifidobacterium* abundance [15]. In vivo and in vitro studies [18, 19] also supported the inhibitory effect of cigarette smoke on the growth of *Bifidobacterium*. However, population-level studies with higher evidence levels for causality are lacking.

Given the essential role of the gut microbiota in the regulation of the central nervous system (CNS) [20], another interesting question is whether smoking behaviors are affected by the gut microbiota. Currently, the microbiota-gut–brain axis, i.e., gut microbiota changes may alter brain function, piqued significant research interest [21]. A recent review summarized the evidence for the presence of bidirectional communications of the axis, and such crosstalk has been linked to major depressive disorder and other psychiatric disorders [22]. Another recent study also identified that abundant genetic signals associated with the gut microbiome were enriched in the genes of neurological functions [23]. In the meantime, the neurological function of the brain was further closely related to tobacco use. Two brain areas, the orbitofrontal cortex and the prefrontal cortex, could interact to turn nicotine cravings on or off [24, 25]. The dopamine reward circuit in the limbic system of the brain was a widely accepted mechanism of tobacco use that withdrawal from nicotine, the main component of cigarettes, will cause a drastic decrease in dopamine secretion. Moreover, animal studies have directly shown that altering the gut microbiome could affect the reward- and stress-related behavior associated with substance abuse, including tobacco [26–28]. Therefore, the gut microbiota has the possibility to affect smoking though the pathways of microbiota-gut-brain-smoking and on the contrary gut microbial homeostasis could be a potential target for addressing tobacco use via improving brain functions [29]. However, the direct links from the microbiome to smoking behaviors which concordance with the gut-brain axis were largely unexplored.

Meanwhile, prior evidence has been found that the manner of communication between the microbiota and the brain involves autonomic nervous system with corresponding neurotransmitters (e.g., γ-aminobutyric acid (GABA), endorphins), bacterial metabolites (typically, short-chain fatty acids) [21], etc. Amino acid metabolites and amino acid-related derivatives are essential sources of most important neurotransmitters. Therefore, associations between the microbiome and smoking could be bridged by relevant metabolites, such as tryptophan (the raw material for serotonin, as known as 5-hydroxytryptamine, 5HT).

Mendelian randomization (MR) is an increasingly used approach to integrate summary data of genome-wide association study (GWAS) to identify causal links between exposures and outcomes. The main reason for its advantage in inferring causality is that MR employs the genetic variants as instrumental variables. MR uses the facts that (1) genetic variants are randomly inherit one allele from each of the father and mother (namely the law of segregation assortment) and (2) alleles will be passed to offspring independently of each other (namely the law of independent assortment). Therefore, MR results are unlikely to be influenced by the environment that might confound the estimated relationship. Recently, the MiBioGen consortium [30] released numerous microbiome abundance-associated loci, offering an unprecedented chance to explore the causality between the gut microbiota and tobacco use. Based on the knowledge above, we hypothesized that the gut microbiome links smoking behaviors and conducted a two-sample bi-directional MR analysis to elucidate the causal association between the gut microbiota and smoking phenotypes and further explore the potential role of several metabolites on these associations.

## Methods

An overview of the analytical plan is shown in Additional file 1: Figure S1.

### Data sources and instrumental variable selection

The data analyzed in this secondary study is publicly available from existing, published GWASs and therefore the ethical approval and informed consent have been obtained by all original studies (Table 1). Detailed information, such as recruitment criteria of population and quality control of genetic data, can be found in the original paper (Table 1). The source data and its related papers were found on PubMed and acquired easily on GWAS Catalog (https://www.ebi.ac.uk/gwas/downloads/summary-statistics). The search terms for summary statistics of gut microbes on PubMed was “gut microbiota” and “genome-wide association study”. The specific terms of smoking phenotypes and metabolites, for example smoking initiation or tryptophan, were directly used to found summary statistics on GWAS Catalog. These GWAS sample populations needed to be predominantly of European descent and largely independent of each other.

**Table 1:** Characteristics of data in this study

Trait		Sample size	Population	Data source (PMID)	Description
Gut microbiome	Phylum	18,340	European (16 cohorts, *N* = 13,266), Middle-Eastern (1 cohort, *N* = 481), East Asian (1 cohort, *N* = 811), American Hispanic/Latin (1 cohort *N* = 1097), African American (1 cohort, *N* = 114) multi-ancestry (4 cohorts, *N* = 2571)	MiBioGen consortium; www.mibiogen.org; (PMID:33462485)	The taxa present in more than 10% of the samples were included
	Class				
	Order				
	Family				
	Genus				
Smoking phenotypes	Age of initiation	341,427	European	GSCAN consortium; https://doi.org/10.13020/3b1n-ff32; (PMID:30643251)	1-SD increase in the age of initiation of regular smoking
	Smoking initiation	1,232,091			Ever smoked regularly compared with never smoked
	Cigarettes per day	337,334			1-SD increase in the number of cigarettes smoked per day
	Smoking cessation	547,219			Current smokers compared with former smokers
	Lifetime smoking	462,690	European	UK Biobank; https://doi.org/10.5523/bris.10i96zb8gm0j81yz0q6ztei23d; (PMID: 31689377)	1-SD increase in the lifetime smoking index was scaled to an individual smoking 20 cigarettes a day for 15 years and quitting 17 years ago, or smoking 60 cigarettes a day for 13 years and quitting 22 years ago
Neurotransmitter-associated or bacterial metabolites	Tryptophan	7824	European	Shin’s Lab; http://metabolomics.helmholtz-muenchen.de/gwas; (PMID: 24816252)	Amino acid (measured by LC/MS pos)
	Tyrosine				Amino acid (measured by LC/MS pos)
	Phenylalanine				Amino acid (measured by LC/MS pos)
	Glutamate				Amino acid (measured by GC/MS)
	Glycine				Amino acid (measured by GC/MS)
	Valerate				Short chain fatty acid (measured by LC/MS neg)

*N* number, *SD* standard deviation, *GSCAN* GWAS & Sequencing Consortium of Alcohol and Nicotine use, *LC/MS* liquid chromatography-mass spectrometry, *GC/MS* gas chromatography-mass spectrometry

The genetic instrument variables (IVs), typically single-nucleotide polymorphisms (SNPs), for the gut microbiota were retrieved from a large-scale GWAS meta-analysis, which contained 18,340 European-dominated participants from 24 separate cohorts with 5,717,754 SNPs after imputation [31, 32]. In the original study, the gut microbiota was categorized into 257 taxa at six taxonomic levels: phylum [p], class [c], order [o], family [f], and genus [g]. Of these, 211 taxa (9 phyla, 16 classes, 20 orders, 35 families, and 131 genera), which were eligible for the mbQTL (microbial quantitative trait locus) mapping analysis, were included in this study. The effect sizes of smoking-related SNPs were acquired from a meta-analyzed GWAS summary association data from 1,232,091 individuals with predominantly European ancestry [30, 33], including age of smoking initiation (a continuous phenotype), smoking initiation (a binary phenotype, ever being a regular smoker), cigarettes per day (a continuous indicator of smoking heaviness), and smoking cessation (a binary phenotype, contrasting current versus former smokers). Of note, lifetime smoking was also included as a comprehensive phenotype, using data from the UK Biobank which recruited 462,690 European ancestry dominated samples [34, 35]. The lifetime smoking is a continuous composite concept of the burden of lifetime exposure to smoking constructed by smoking initiation/cessation, smoking heaviness, and smoking duration.

Furthermore, we also sought to explore the potential role of neurotransmitters in the biological pathway of the microbiota to smoking. After a systematic literature search in PubMed (we only considered the metabolites whose serum levels were commonly measured in regular metabolomic studies), we identified several important neurotransmitter-associated metabolites (including tryptophan, tyrosine, phenylalanine, glutamate, glycine) that may closely relate to brain function, especially substance use disorder, which subsequently affect smoking [36–39]. In addition, we also considered an important group of bacterial metabolites (i.e., short-chain fatty acids). We extracted genetic data for these specific human blood metabolites (i.e., tryptophan, tyrosine, phenylalanine, glutamate, glycine, and valerate) from a GWAS comprising 7824 European adult individuals [40, 41]. Specifically, tryptophan, tyrosine, phenylalanine, and glutamate are closely related to neurotransmitters, respectively, 5-HT, dopamine, endorphins, and GABA. Glycine itself is a kind of neurotransmitter [42]. Valerate belongs to the short-chain fatty acids, which could regulate the blood–brain barrier, myelin formation, vagal excitability, and microglia maturation [43].

**Fig. 1:**
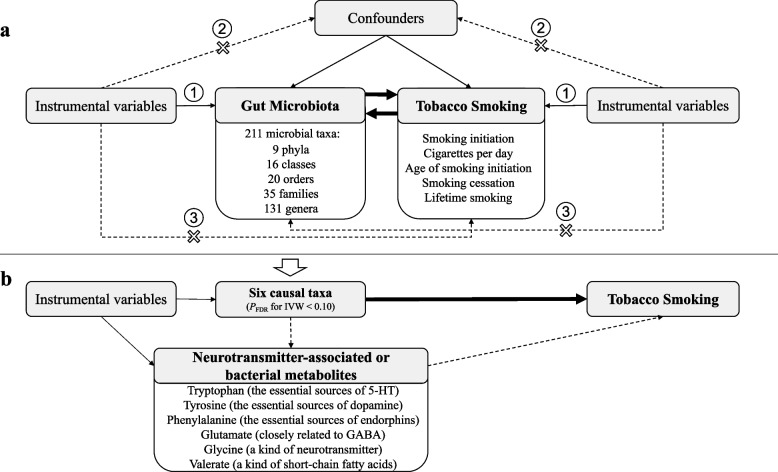
Schematic representation of the **a** two-sample bi-directional Mendelian randomization analysis and **b **multivariable Mendelian randomization analysis. MR was used to evaluate the causal links between 211 microbial taxa and five smoking phenotypes with or without considering neurotransmitter-associated or bacterial metabolites. Three key assumptions of MR: (1) genetic variants must be associated with exposures, (2) genetic variants must not be associated with confounders, and (3) genetic variants must affect outcomes only through exposures, not through other pathways

The selection of IVs, the key to ensure the accuracy and robustness of the causal inferences, should meet MR’s three key assumptions (Fig. 1). Then, the following steps were performed. Firstly, palindromic variants with minor allele frequency greater than 0.4 were excluded; secondly, variants and their alleles were harmonized between the GWAS results of exposure and outcome; thirdly, independent SNPs (LD *r*^2^ < 0.01 and clumping distance = 250 kb, based on the European-based 1000 Genome Projects reference panel) were selected at a compromised significant level (1 × 10^−6^) due to the relatively small sample size for mbQTL identification. To mitigate the effect of weak IV bias, the regular genome-wide significance (5 × 10^−8^) was retained as a sensitivity analysis.

The usage and interpretation of our MR study adhere to the STROBE-MR (Strengthening the Reporting of Observational Studies in Epidemiology-Mendelian Randomization) checklist [44] (Additional file 1: Table S1) and the critical appraisal checklist proposed by Davies et al. [45] (Additional file 1: Table S2).

### Statistical analysis

The two-sample MR study was incorporated to evaluate the causal links between 211 microbial taxa and five smoking phenotypes. The list of covariates varies between original GWASs, but always included sex and age. The details can be found in the original studies. Causal effects were estimated by five high-efficiency methodologies: the multiplicative random-effects model for the inverse-variance weighted (IVW) method (as the main MR method) [46], a constrained maximum likelihood and model averaging-based MR method (cML-MA, also as the main MR method) [47], weighted median method (sensitivity analysis) [48], MR-Egger regression (sensitivity analysis) [49], and MR pleiotropy residual sum and outlier (MR-PRESSO) method (sensitivity analysis) [50]. Different approaches yield valid evidence under different assumptions. cML-MA, which without relying on the InSIDE assumption, was also applied as a complementary method in our study to control correlated and uncorrelated pleiotropic effects [47]. In addition, the Cochran’s Q test in IVW was adopted to test the heterogeneity among SNPs included in each analysis. The intercept from MR-Egger method was used to assess the Instrument Strength Independent of Direct Effect (InSIDE) assumption which assumes that the horizontal pleiotropic effects are independent of the variant-exposure associations. *P*_intercept_ < 0.05 suggests the existence of horizontal pleiotropy. MR-PRESSO global test was also used to evaluate overall horizontal pleiotropy. Meanwhile, MR-PRESSO corrected for horizontal pleiotropy by outlier removal. The above series of analyses was repeated to explore the causality in the other way, i.e., the impacts of tobacco use on gut microbial composition. Finally, as an attempt to uncover possible vertical pleiotropic pathways that could arise from specific serum metabolites, the multivariable MR analyses including MVMR-IVW and MVMR-Egger were performed to estimate the causal effect of specific gut microbes on smoking after adjusting for six neurotransmitter-associated or bacterial metabolites simultaneously and separately. Parameter setting was the same as for univariate MR.

MR analyses were performed using the “TwoSampleMR” (version 0.5.6), “MRcML” (version 0.0.0.9), and “MendelianRandomization” (version 0.6.0) packages in R (version 4.1.2) [50, 51]. The statistical significance of the MR effect estimate was defined as a false discovery rate (FDR) of < 10%, where the Benjamini–Hochberg procedure was used to correct for the number of taxa tested, accounting for multiple comparisons.

## Results

### Causal effect of smoking on gut microbiota

To understand the consequences of smoking behaviors (including smoking initiation, cigarette per day, age of initiation, smoking cessation, and lifetime smoking) on the abundance of the gut microbiome, two-sample MR tests were performed. We tested the potential causality from smoking-related traits to all available gut taxa. Detailed significant results for the causal relationship from smoking phenotypes to gut microbial taxa are shown in Table 2.

**Table 2:** Significant MR results of causal links between gut microbiome and smoking phenotypes by using IVW method and cML-MA method

Exposure	Outcome	No. SNP	Methods	*β*	SE	*P*_IVW_	*P*_FDR_	Horizontal pleiotropy *P*_for Egger intercept_	Heterogeneity *P*_for Cochran’s Q_
Phylum *Actinobacteria*	Age of initiation	6	IVW	0.051	0.019	7.74e-03	0.079	0.466	0.386
			cML-MA	0.053	0.020	7.99e-3	0.082		
Order *Bifidobacteriales*		7	IVW	0.050	0.016	1.73e-03	0.024	0.098	0.714
			cML-MA	0.050	0.015	9.45e-4	0.013		
Family *Bifidobacteriaceae*		7	IVW	0.050	0.016	1.73e-03	0.024	0.098	0.714
			cML-MA	0.050	0.015	9.45e-4	0.013		
Genus *Bifidobacterium*		7	IVW	0.049	0.016	1.79e-03	0.024	0.095	0.725
			cML-MA	0.050	0.015	9.59e-4	0.013		
Phylum *Actinobacteria*	Cigarettes per day	6	IVW	-0.066	0.024	5.31e-03	0.092	0.357	0.139
			cML-MA	-0.063	0.021	3.28e-3	0.050		
Class *Actinobacteria*		8	IVW	-0.053	0.018	3.70e-03	0.092	0.446	0.084
			cML-MA	-0.050	0.016	1.93e-3	0.050		
Order *Bifidobacteriales*		7	IVW	-0.048	0.019	1.12e-02	0.092	0.455	0.077
			cML-MA	-0.043	0.017	9.67e-3	0.050		
Family *Bifidobacteriaceae*		7	IVW	-0.048	0.019	1.12e-02	0.092	0.455	0.077
			cML-MA	-0.043	0.017	9.67e-3	0.050		
Genus *Bifidobacterium*		7	IVW	-0.048	0.018	8.83e-03	0.092	0.552	0.074
			cML-MA	-0.043	0.016	7.69e-3	0.050		
Phylum *Actinobacteria*	Lifetime smoking	6	IVW	-0.023	0.009	8.43e-03	0.056	0.398	0.803
			cML-MA	-0.02	0.009	9.09e-3	0.042		
Class *Actinobacteria*		7	IVW	-0.019	0.007	5.79e-03	0.056	0.770	0.469
			cML-MA	-0.019	0.007	6.00e-3	0.042		
Order *Bifidobacteriales*		6	IVW	-0.023	0.008	5.32e-03	0.056	0.468	0.152
			cML-MA	-0.024	0.007	7.71e-4	0.012		
Family *Bifidobacteriaceae*		6	IVW	-0.023	0.008	5.32e-03	0.056	0.468	0.152
			cML-MA	-0.024	0.007	7.71e-4	0.012		
Genus *Bifidobacterium*		6	IVW	-0.023	0.008	7.00e-03	0.056	0.385	0.161
			cML-MA	-0.023	0.007	9.08e-4	0.012		
Genus *Peptococcus*		4	IVW	-0.019	0.007	7.30e-03	0.056	0.371	0.540
			cML-MA	-0.019	0.007	9.49e-3	0.042		
Age of initiation	Genus *Eisenbergiella*	24	IVW	-1.062	0.250	2.21e-05	0.005	0.476	0.320
			cML-MA	-1.101	0.248	8.77e-06	0.002		
	Genus *Lactococcus*	23	IVW	1.135	0.317	3.45e-04	0.036	0.222	0.241
			cML-MA	1.188	0.297	6.39e-05	0.007		
Smoking initiation	Order *Pasteurellales*	287	IVW	-0.328	0.097	7.40e-04	0.039	0.797	0.725
			cML-MA	-0.344	0.099	5.42e-4	0.023		
	Family *Pasteurellaceae*	287	IVW	-0.328	0.097	7.40e-04	0.039	0.797	0.533
			cML-MA	-0.344	0.099	5.42e-4	0.023		
	Family *Christensenellaceae*	291	IVW	-0.278	0.072	1.18e-04	0.025	0.103	0.725
			cML-MA	-0.284	0.074	1.35e-4	0.023		
	Genus *ChristensenellaceaeR*	291	IVW	-0.267	0.073	2.75e-04	0.029	0.068	0.775
			cML-MA	-0.272	0.075	3.22e-4	0.023		
	Genus *Haemophilus*	287	IVW	-0.321	0.099	1.15e-03	0.041	0.554	0.827
			cML-MA	-0.339	0.101	7.91e-4	0.028		
	Genus *Intestinimonas*	290	IVW	0.265	0.090	3.15e-03	0.095	0.801	0.110
			cML-MA	0.273	0.088	1.90e-3	0.057		
	Genus *Romboutsia*	290	IVW	-0.279	0.085	1.06e-03	0.041	0.611	0.026
			cML-MA	-0.294	0.081	2.84e-4	0.023		
Lifetime smoking	Class *Coriobacteriia*	372	IVW	0.242	0.069	4.72e-04	0.020	0.241	0.971
			cML-MA	0.248	0.070	3.98e-4	0.016		
	Order *Coriobacteriales*	372	IVW	0.242	0.069	4.72e-04	0.020	0.241	0.971
			cML-MA	0.248	0.070	3.98e-4	0.016		
	Family *Coriobacteriaceae*	372	IVW	0.242	0.069	4.72e-04	0.020	0.241	0.971
			cML-MA	0.248	0.070	3.98e-4	0.016		
	Genus *Catenibacterium*	328	IVW	0.505	0.170	2.98e-03	0.090	0.333	0.478
			cML-MA	0.516	0.173	2.80e-3	0.085		
	Genus *RuminococcaceaeNK4A214*	371	IVW	-0.261	0.074	4.48e-04	0.020	0.996	0.513
			cML-MA	-0.266	0.076	4.67e-4	0.016		
	Genus *RuminococcaceaeUCG005*	371	IVW	-0.237	0.076	1.91e-03	0.067	0.887	0.072
			cML-MA	-0.269	0.074	3.05e-4	0.016		
	Genus *Eubacterium xylanophilum*	370	IVW	-0.308	0.080	1.08e-04	0.020	0.035	0.802
			cML-MA	-0.317	0.081	8.94e-05	0.016		

*MR* Mendelian randomization, *IVW* inverse-variance weighted, *cML-MA* constrained maximum likelihood and model averaging-based MR method, *No.SNP* number of single-nucleotide polymorphism (SNP), *SE* standard error, *P*_*FDR*_* P*-value corrected by false discovery rate (FDR) across tested taxa

**Fig. 2:**
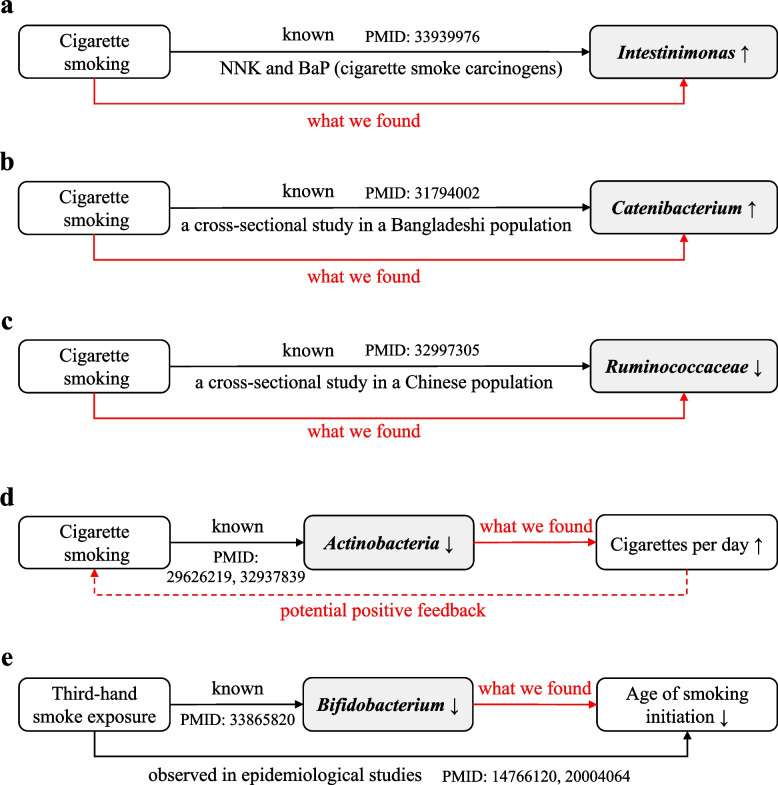
The summary of the key findings in the univariable MR study**.** Combining with existing evidence, we consistently found the causal effect of smoking on **a** *Intestinimonas*, **b** *Catenibacterium*, **c** *Ruminococcaceae*, and provided novel insights regarding **d** the reward loop of smoking heaviness, **e** the influence of parental smoking on childhood smoking. Black solid arrows indicate known evidence, red solid arrows indicate what we found in this study, and red dashed arrow indicates potentially deducible conclusion

The results of IVW analyses showed that the genetic liability for smoking initiation had a causal contribution to an increased abundance of *Intestinimonas[g]* (Beta ± SE: 0.265 ± 0.090, *P* = 3.15e − 03), which was in line with the evidence from a mice model showing that exposure to the major cigarette smoke carcinogens (NNK plus BaP) could elevate fecal level of *Intestinimonas* [52] (Fig. 2a). We also found that increased genetically predicted lifetime smoking was significantly related to higher abundance of *Catenibacterium[g]* (Beta ± SE: 0.505 ± 0.170, *P* = 2.98e − 03) as well as lower abundance of *RuminococcaceaeNK4A214[g]* (Beta ± SE: − 0.261 ± 0.074, *P* = 4.48e − 04) and *RuminococcaceaeUCG005[g]* (Beta ± SE: − 0.237 ± 0.076, *P* = 1.91e − 03), corroborating previous observational findings from two cross-sectional studies based on a Bangladeshi population [53] and a Chinese population [54], respectively (Fig. 2b,c). The MR results also suggested that smoking initiation was causally associated with the abundance of *Pasteurellales[o]* (Beta ± SE: − 0.328 ± 0.097, *P* = 7.40e − 04), *Pasteurellaceae[f]* (Beta ± SE: − 0.328 ± 0.097, *P* = 7.40e − 04), *Christensenellaceae[f]* (Beta ± SE: − 0.278 ± 0.072, *P* = 1.18e − 04), *ChristensenellaceaeR[g]* (Beta ± SE: − 0.267 ± 0.073, *P* = 2.75e − 04), and *Romboutsia[g]* (Beta ± SE: − 0.279 ± 0.085, *P* = 1.06e − 03). A higher genetically predicted age of smoking initiation was causally related to a higher abundance of *Lactococcus[g]* (Beta ± SE: 1.135 ± 0.317, *P* = 3.45e − 04), but a lower abundance of *Eisenbergiella[g]* (Beta ± SE: − 1.062 ± 0.250, *P* = 2.21e − 05). Neither horizontal pleiotropy nor heterogeneity (among IVs) was detected at statistically significant levels (all *P*_for Egger intercept_ > 0.05, most of the *P*_for PRESSSO global test_ > 0.05, and all *P*_for Cochran’s Q_ > 0.05). The results estimated by cML-MA method was highly consistent with the estimates using IVW. The FDR adjusted *P*-value and the family-wised corrected *P*-value could be found in Additional file 2: Table S4.

### Causal effect of gut microbiota on smoking

The original GWAS involving 18,340 individuals from 24 cohorts provided summary statistics for 211 microbial taxa. Of them, 41 taxa providing greater than or equal to three IVs were included in this MR for testing the potential causal effect of the gut microbiome on smoking behaviors (40 taxa for lifetime smoking). Estimated by IVW test, six taxa were identified, after FDR correction, to reach a statistical significance. Concordant results were observed using cML-MA method (shown in Table 2).

The MR analysis revealed that the abundance of *Actinobacteria[p]* (Beta ± SE: − 0.066 ± 0.024, *P* = 5.31e − 03), *Actinobacteria[c]* (Beta ± SE: − 0.053 ± 0.018, *P* = 3.70e − 03), *Bifidobacteriales[o]* (Beta ± SE: − 0.048 ± 0.019, *P* = 1.12e − 02), *Bifidobacteriaceae[f]* (Beta ± SE: − 0.048 ± 0.019, *P* = 1.12e − 02), and *Bifidobacterium[g]* (Beta ± SE: − 0.048 ± 0.018, *P* = 8.83e − 03) were negatively associated with the number of cigarettes smoked per day. Increased abundance of *Actinobacteria[p]* (Beta ± SE: 0.051 ± 0.019, *P* = 7.74e − 03), *Bifidobacteriales[o]* (Beta ± SE: 0.050 ± 0.016, *P* = 1.73e − 03), *Bifidobacteriaceae[f]* (Beta ± SE: 0.050 ± 0.016, *P* = 1.73e − 03), and *Bifidobacterium[g]* (Beta ± SE: 0.049 ± 0.016, *P* = 1.79e − 03) lead to later smoking initiation. When considering lifetime smoking as an outcome, the results showed a trend similar to that of cigarettes per day analysis (detailed effect estimates are shown in Table 1). The sensitivity analyses did not show clear evidence of potential horizontal pleiotropy (all *P*_for Egger intercept_ > 0.05, most of the *P*_for PRESSSO global test_ > 0.05). Heterogeneity was not found among SNPs (all *P*_for Cochran’s Q_ > 0.05).

Another two key findings are displayed in Fig. 2d and e, indicating potential mechanisms of the gut-brain axis. All causal-effect estimates, including the sensitivity analyses, between 211 microbial taxa and five smoking phenotypes analyzed in this MR are presented in Additional file 2: Table S4. All IVs used in our study are provided in Additional file 2: Table S5. Additional visualizations of the results, including scatter plot, forest plot, and leave-one-out plot can be found in Additional file 1: Figure S2-S5. Moreover, the MR results evaluated under two instrumental variable selection thresholds (1e − 6 vs. 5e − 8) were presented in Additional file 1: Table S3, which indicated very limited difference on *β*-coefficients (Pearson r_1e-6v.s.5e-8_ = 0.99, *P* < 0.001).

### The effect of neurotransmitter-associated metabolites on observed associations

Considering the possible contribution of serum metabolites on the progress from the gut microbiota to smoking, we used multivariable MR for observed significant associations (the results in Table 2) with six neurotransmitter-associated or bacterial metabolites (tryptophan, tyrosine, phenylalanine, glutamate, glycine, and valerate) adjusted. The multivariable MR results are reported in Table 3.

**Table 3:** Multivariable MR results of causal links between gut microbiome and smoking phenotypes after adjusting for specific serum metabolites

Exposure	Outcome	Adjustment of metabolites	Method	*β*	SE	*P*	Horizontal pleiotropy *P*_for Egger intercept_
Phylum *Actinobacteria*	Age of initiation	Six metabolites	MVMR-IVW	0.044	0.018	0.013	-
			MVMR-Egger	0.048	0.023	0.040	0.813
Order *Bifidobacteriales*		Six metabolites	MVMR-IVW	0.029	0.015	0.044	-
			MVMR-Egger	0.045	0.018	0.011	0.124
Family *Bifidobacteriaceae*		Six metabolites	MVMR-IVW	0.029	0.015	0.044	-
			MVMR-Egger	0.045	0.018	0.011	0.124
Genus *Bifidobacterium*		Six metabolites	MVMR-IVW	0.027	0.014	0.062	-
			MVMR-Egger	0.045	0.017	0.009	0.071
Phylum *Actinobacteria*	Cigarettes per day	Six metabolites	MVMR-IVW	-0.067	0.039	0.089	-
			MVMR-Egger	-0.126	0.050	0.012	0.062
Class *Actinobacteria*		Six metabolites	MVMR-IVW	-0.058	0.032	0.074	-
			MVMR-Egger	-0.084	0.040	0.036	0.267
Order *Bifidobacteriales*		Six metabolites	MVMR-IVW	-0.063	0.032	0.046	-
			MVMR-Egger	-0.106	0.038	0.006	0.055
Family *Bifidobacteriaceae*		Six metabolites	MVMR-IVW	-0.063	0.032	0.046	-
			MVMR-Egger	-0.106	0.038	0.006	0.055
Genus *Bifidobacterium*		Six metabolites	MVMR-IVW	-0.061	0.031	0.050	-
			MVMR-Egger	-0.112	0.037	0.003	0.018
Phylum *Actinobacteria*	Lifetime smoking	Six metabolites	MVMR-IVW	-0.018	0.009	0.040	-
			MVMR-Egger	-0.020	0.011	0.084	0.818
Class *Actinobacteria*		Six metabolites	MVMR-IVW	-0.009	0.008	0.242	-
			MVMR-Egger	-0.011	0.009	0.262	0.755
Order *Bifidobacteriales*		Six metabolites	MVMR-IVW	-0.015	0.008	0.045	-
			MVMR-Egger	-0.018	0.010	0.064	0.685
Family *Bifidobacteriaceae*		Six metabolites	MVMR-IVW	-0.015	0.008	0.045	-
			MVMR-Egger	-0.018	0.010	0.064	0.685
Genus *Bifidobacterium*		Six metabolites	MVMR-IVW	-0.014	0.008	0.060	-
			MVMR-Egger	-0.020	0.009	0.035	0.317
Genus *Peptococcus*		Six metabolites	MVMR-IVW	-0.005	0.008	0.502	-
			MVMR-Egger	-0.006	0.010	0.550	0.795
Class *Actinobacteria*	Lifetime smoking	Tryptophan	MVMR-IVW	-0.012	0.008	0.115	-
			MVMR-Egger	-0.015	0.009	0.101	0.553
		Tyrosine	MVMR-IVW	-0.013	0.013	0.316	-
			MVMR-Egger	-0.039	0.020	0.051	0.102
		Phenylalanine	MVMR-IVW	-0.016	0.006	0.006	-
			MVMR-Egger	-0.030	0.022	0.173	0.501
		Glutamate	MVMR-IVW	-0.018	0.006	0.001	-
			MVMR-Egger	-0.012	0.017	0.462	0.710
		Glycine	MVMR-IVW	-0.015	0.005	0.004	-
			MVMR-Egger	-0.018	0.012	0.145	0.775
		Valerate	MVMR-IVW	-0.009	0.007	0.218	-
			MVMR-Egger	-0.047	0.030	0.121	0.185
Genus *Peptococcus*	Lifetime smoking	Tryptophan	MVMR-IVW	-0.005	0.008	0.503	-
			MVMR-Egger	-0.002	0.010	0.835	0.663
		Tyrosine	MVMR-IVW	-0.013	0.023	0.558	-
			MVMR-Egger	-0.044	0.021	0.038	0.013
		Phenylalanine	MVMR-IVW	-0.022	0.012	0.071	-
			MVMR-Egger	-	-	-	-
		Glutamate	MVMR-IVW	-0.022	0.012	0.072	-
			MVMR-Egger	-0.063	0.068	0.355	0.526
		Glycine	MVMR-IVW	-0.020	0.008	0.009	-
			MVMR-Egger	-0.024	0.011	0.035	0.549
		Valerate	MVMR-IVW	-	-	-	-
			MVMR-Egger	-	-	-	-

*MR* Mendelian randomization, *MVMR* multivariable Mendelian randomization, *SE* standard error, *IVW* inverse-variance weighted

When adjusting the six metabolites together using MVMR-IVW, we found a noticeable increase in *P*-value for causal effect of *Actinobacteria[c]* (*P* = 0.242) and *Peptococcus[g]* (*P* = 0.502) on lifetime smoking, while the majority of results remained robust. Another change worth mentioning is that, by making rough comparisons, relatively lower *β*-coefficient (absolute value) for the associations between the gut microbiota and age of initiation were observed compared with the results without adjustment for the metabolites. Subsequently, focused on *Actinobacteria[c]* and *Peptococcus[g]*, we implemented adjustment of one metabolite at a time. Signals of decline in significance of association were detected for tryptophan (*P* = 0.115 for *Actinobacteria[g]*; *P* = 0.503 for *Peptococcus[g]*), tyrosine (*P* = 0.316 for *Actinobacteria[g]; P* = 0.558 for *Peptococcus[g]*), and valerate (*P* = 0.218 for *Peptococcus[g]*). Additionally, the results of MVMR-Egger indicated that our multivariable MR estimates were unlikely biased by pleiotropy (most of *P*_for MVMR-Egger intercept_ > 0.05).

**Fig. 3:**
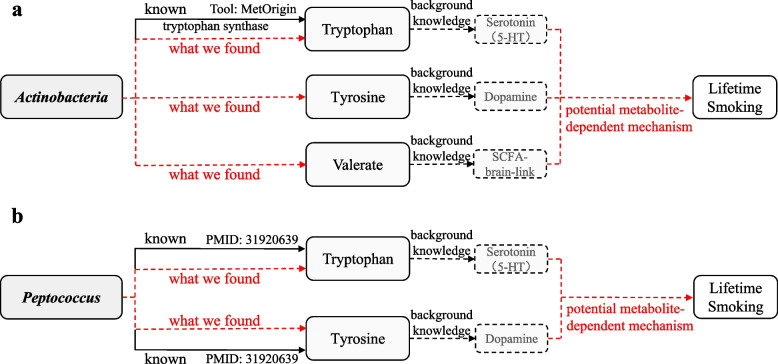
The summary of the key findings in the multivariable MR study. Combining with existing evidence and biological mechanisms, we consistently found that tryptophan, tyrosine, and/or valerate may take part in the action pathways from **a** *Peptococcus* and **b** *Actinobacteria* to smoking. Black solid arrows indicate known evidence, black dashed arrows indicate biological hypothesis we introduced earlier, and red dashed arrow indicates potentially deducible conclusion. The bold dashed boxes are neurotransmitters that are closely related to corresponding metabolites and are likely involved in the mechanism by which smoking responds to the gut microbiota in a metabolite-dependent manner

The intriguing finding was presented in Fig. 3, implying the role of metabolites in the action pathway from the gut microbiota to smoking.

## Discussion

To our best knowledge, this work is among the first to systematically evaluate the causal relationships between the gut microbiota and tobacco use from a genetic perspective. This two-sample MR study gave reasonably strong evidence that genetically predicted abundance of specific gut microbes play non-negligible roles in the occurrence and progression of cigarette smoking, in which, metabolites may be participating. As for the other direction, the MR confirmed and strengthened the role of smoking on the gut microbiota. Leveraging the power of molecular genetic markers as instrumental variables, the MR approaches largely avoided the interference of confounders (e.g., socioeconomic position, culture) and reversed causality which make regular observational study vulnerable [45].

As mentioned in the introduction, the theory of the microbiota-gut-brain communication hints at a possible influence of the gut microbiota on smoking. Nevertheless, few studies had directly explored this theme. Leveraging the large-scale GWAS data sources, our MR study filled this knowledge gap from a novel angle. (1) Previous studies indicated that smoking would decrease the abundance of *Actinobacteria* while our results found that a lower abundance of *Actinobacteria* may cause an increase in the number of cigarettes smoked per day, i.e., worse smoking status. More severe smoking conditions may in turn cause a further diminishment in *Actinobacteria* abundance, implying a potential positive feedback effect [10, 55]. This might partially explain why smokers tend to increase the tobacco use. (2) In addition, observational research showed that, compared to infants from non-smoking families, those from smoking households had lower intestinal flora diversity and abundance, with *Bifidobacterium* in particular [56]. Interestingly, we found a lower abundance of *Bifidobacterium* may induce an earlier age of smoking initiation. While conventional wisdom has expounded that early smoking in children may result from early exposure to third-hand smoke or imitation of father’s smoking behavior [57, 58], our study provides new insights that the early smoking initiation may be proportionally explained by gut flora. Regulating the gut microbiota, such as probiotic intervention, might be an option to redeem bad effects caused by premature smoke exposure.

Rather than just concerning the causality between the gut microbiota and smoking, we also considered the possible involvement of metabolites in this process. (1) Our results suggested an attenuated significance of association between *Peptococcus* and smoking after adjusting tryptophan and/or tyrosine, implying a potential metabolite-dependent mechanism of the microbiota on smoking that these two amino acids drove. Wen’s study, from another angle that using metabolomics and 16S rRNA gene sequencing analyses in the rat model, proved the correlation between *Peptococcus* and key metabolic pathways, also including tryptophan metabolism and tyrosine metabolism [59]. (2) MetOrigin is a bioinformatics tool, aiming to identify which bacteria and how they participate in certain metabolic reactions [60]. A similar implication in our multivariable MR analysis that tryptophan may modify the effects of *Actinobacteria* on smoking was also somewhat corroborated in this platform by a simple quick search, which supported the relationship between *Actinobacteria* and tryptophan synthase.

There is growing evidence, albeit some indirect, providing possible biological explanations for the mechanisms of commensal gut microbiota on smoking, particularly probiotics such as *Bifidobacterium*. (1) The vagus nerve is thought to be a major modulatory constitutive communication pathway between the intestinal bacteria and the brain. *Bifidobacterium longum* have been found, via the vagus nerve, to send signals to the brain, leading to the secretion of a higher level of dopamine [61]. Since dopamine is related to the brain's reward function, higher levels of dopamine will offset the euphoria of smoking or the pain of quitting, thereby reducing smoking addiction [62]. (2) Neurotransmitters probably mediate the influences of the intestinal microbiome on smoking. For instance, *Bifidobacterium* was reported to promote serotonin (5-HT) biosynthesis in colonic enterochromaffin cells by activating the CGA/ADRα2A cascade signal and regulating the TRP/TPH-OR pathways [63, 64]. 5-HT has been the therapeutic target for addiction to alcohol, cocaine, or drug, so it may also be for smoking [65]. Other neurotransmitters with similar functions and previously shown to be influenced by *Bifidobacterium* also include GABA [66] and noradrenaline [67]. (3) The close link of metabolites (e.g., short-chain fatty acids [68], metabolite acetate [69]) or components (e.g., peptidoglycan [PGN]) of *Bifidobacterium* with the CNS may explain its effect on smoking. Short-chain fatty acids are relevant to the morphology and function of microglia [70], and metabolite acetate has therapeutic potential to prevent cognitive impairment [69]. PNG can penetrate the blood-brain barrier, entering the brain, and communicating with the PGN-sensing molecules (Pglyrp2) in the amygdala [71, 72]. Accordingly, changes in metabolite levels resulting from gut flora dysbiosis make an unavoidable effect on CNS, releasing fear- or anxiety-like emotions or triggering depression, subsequently elevating the risk of smoking initiation or failure to smoking cessation [73–75]. The above biological evidence also explains, to some extent, why the relationship between the gut microbiota and smoking may be modified after adjusting for specific amino acids or short-chain fatty acids. A point worth noting is that these potential mechanisms are not fully evidenced. In addition, there is a non-negligible gap between nicotine cravings and the complex smoking behaviors/pattern observed at the population level. Future studies on the gut microbiome and smoking behaviors are anticipated. Certainly, before moving forward, specialized mechanistic investigations are needed to understand the distinct roles of individual taxa, as most of the currently available mechanistic explanations remain at the generalized whole-gut microbial level.

In the other direction, our findings strengthened and extended existing observational evidence, suggesting that tobacco smoking could disrupt the homeostasis of the intestinal microbiota. (1) Our study supported that initiation of smoking could increase *Intestinimonas* abundance, which showed consistency with the results obtained in a previous experimental study. Qu and colleagues observed an elevated level of *Intestinimonas* after exposure to NNK plus BaP in mice [52]. Notably, NNK and BaP, the products of smoking, are the major risk elements for inducing cellular carcinogenesis of lung cancer [76]. (2) The MR results confirmed the roles of smoking for a higher abundance of *Catenibacterium* and a lower abundance of *Ruminococcaceae* which were observed from two cross-sectional studies. In a Bangladeshi population, a study exhibited that the relative abundance of *Catenibacterium* was significantly higher in current smokers compared with never-smokers, showing a dose-response relationship with packs of cigarettes smoked per day [53]. Enrolling 116 healthy male subjects from China, an observational study revealed that smoking could lower the abundance of *Ruminococcaceae*, which was independent of BMI and age [54]. Importantly, MR design allows for more reliable results with the highest evidence hierarchy other than randomized controlled trials (RCT) [45]. (3) In addition, Wang et al. reported that cigarette smoking significantly reduced the level of *Lactococcus* [77]. The current MR study further pointed out that the younger the year of smoking initiation, the greater this reduction. (4) Apart from the above, there also appeared several significant evidence for the effect of smoking on *Eisenbergiella*, *Pasteurellaceae*, *Christensenellaceae*, *Haemophilus*, *Romboutsia*, and *Coriobacteriaceae*, which were rarely addressed or not clearly understood before. (5) Nevertheless, it was noteworthy that for some microbial taxa, such as *Bifidobacterium* and *Actinobacteria*, the existing literature reported the impact of smoking on these taxa, while our work did not provide corresponding strong causal evidence, although most of the effect estimates were consistent in the direction. The main mechanisms by which smoking affects the gut microbiota include the following: raising the pH of the intestinal environment [18], inducing chronic low-grade inflammation or inflammation-related diseases [78], as well as promoting oxidative stress [79].

Several limitations of our study should be acknowledged. Firstly, to reduce the potential effect of weak IV bias, we applied a stricter *P*-value cutoff (1e − 06), compared with 1e − 05 which was used in the original paper [31] and another recent paper [80]. Thus, it may result in insufficient statistical power, a critical reason for false negatives. Because of the large number of microbial taxa, as well as the hierarchical structure (the abundance could be highly correlated for a microbial strain), and correlations among smoking phenotypes, the multiple comparison adjustment, especially global multiple corrections, may be excessive, further affecting the false negative. Therefore, causality cannot be completely ruled out in negative results, which should be treated with caution. Secondly, since the majority of participants in the GWAS of tobacco use were ancestrally Europeans, extrapolation of the results in the present study to other ethnic groups might be limited. Thirdly, although most of the participants of the gut microbial GWAS were ancestrally Europeans, the ethnic proportion was not perfectly matched between the two samples (i.e., the exposure GWAS and the outcome GWAS dataset), which may result in some levels of inconsistency in LD correlations. Fourthly, smoking is predominantly prevalent among men, and the composition of the gut microbiota also somewhat varies by gender. However, our work cannot analyze the two genders separately. Likewise, the estimates of a lifetime effect of the gut microbiota on smoking provided by MR cannot deliver much clinical meaningful for age-specific interventions. The limited sample size may also prevent us from providing a sufficiently precise estimate as well as 95% confidence intervals for clinical practice. It would be helpful to perform a gender- or age-specific MR analysis especially with larger sample size in future endeavors. Finally, the metabolites analyzed in multivariable MR were detected in human serum. We think that more appropriate and direct information may be generated from fecal samples, but unfortunately, this kind of data is currently lacking. Additionally, direct analysis of all metabolites may leave the hypothesis without sufficient biological evidence, whereas a biologically informed selection may reduce significant findings. Of a certainty, there still exist other neurotransmitters such as norepinephrine as well as other more important short-chain fatty acids such as propionate and butyrate, but the summary data of itself or its related metabolites is also lacking.

## Conclusions

Leveraging the publicly available genetic databases, bidirectional causal links between specific intestinal microbes and cigarette smoking were identified. Taking together the existing evidence, potential mechanisms including a positive feedback loop of smoking and the potential role of neurotransmitter-associated metabolic biomarkers therein were revealed. Our study highlighted the hazards of tobacco use for gut flora dysbiosis and shed light on the potential role of specific gut microbiota for tobacco use behaviors.

## Supplementary Information

**Additional file 1: Table S1.** Self-inspection results of STROBE-MR checklist of recommended items to address in reports of Mendelian randomization studies. **Table S2.** Self-inspection results of critical appraisal checklist proposed by Davies et al. for evaluating Mendelian randomization studies. **Table S3.** The MR results of causal links between gut microbiome and smoking phenotypes by using IVW method under two instrumental variable selection thresholds. **Figure S1.** Overview of the analytical plan and main findings. **Figure S2.** Scatter plot of associations between genetic variants and Actinobacteria[p] versus between genetic variants and Cigarettes Per Day. The slope of each line represents the causal effect estimate using the corresponding MR analysis model, and the intercept can be interpreted as an estimate of the average horizontal pleiotropic effect across the genetic variants. **Figure S3.** Forest plot of individual SNP estimates and summary estimates for the causal associations between Actinobacteria[p] abundance and Cigarettes Per Day. **Figure S4.** Leave-one-out plot to assess if a single SNP drives the causal association between Actinobacteria[p] abundance and Cigarettes Per Day. **Figure S5.** Funnel plot of MR estimation for the causal association between Actinobacteria[p] abundance and Cigarettes Per Day.

**Additional file 2: Table S4.** All results of causal links between gut microbiome and smoking phenotypes. **Table S5.** Instrumental variables used in our study for univariate MR and multivariable MR, respectively.

## Data Availability

The data used in this study is freely available for download in the MiBioGen repository, www.mibiogen.org, GSCAN repository, https://doi.org/10.13020/3b1n-ff32, UK Biobank repository, https://doi.org/10.5523/bris.10i96zb8gm0j81yz0q6ztei23d, and the Metabolomics GWAS repository, http://metabolomics.helmholtz-muenchen.de/gwas, respectively. Availability of code The actual code used to run the analyses described in this study is available at the github site at https://github.com/zdangm/smoking_microbiome.

## Abbreviations

5HT: 5-Hydroxytryptamine, as known as serotonin
BaP: Benzo[a]pyrene
BMI: Body mass index
cML-MA: A constrained maximum likelihood and model averaging-based MR method
CNS: Central nervous system
FDR: False discovery rate
GABA: γ-Aminobutyric acid
GWAS: Genome-wide association study
InSIDE: Instrument Strength Independent of Direct Effect
IV: Instrument variable
IVW: Inverse-variance weighted
LD: Linkage disequilibrium
mbQTL: Microbial quantitative trait locus
MR: Mendelian randomization
MR-PRESSO: MR pleiotropy residual sum and outlier
MVMR: Multivariable Mendelian randomization
NNK: Nitrosamines 4-(methylnitrosamino)-1-(3-pyridyl)-1-butanone
Pglyrp2: PGN-sensing molecules
PGN: Peptidoglycan
RCT: Randomized controlled trial
SNP: Single-nucleotide polymorphism
