# Supplementary material for: Cross-talks between gut microbiota and tobacco smoking: a two-sample Mendelian randomization study
Source: BMC Med. 2023 Apr 28;21:163. doi: 10.1186/s12916-023-02863-1 (PMC10148467; doi:10.1186/s12916-023-02863-1)

**Supplementary files**

**Table S1**: Self-inspection results of STROBE-MR checklist of recommended items to address in reports of Mendelian randomization studies.

**Table S2**: Self-inspection results of critical appraisal checklist proposed by Davies et al. for evaluating Mendelian randomization studies.

**Table S3**: The MR results of causal links between gut microbiome and smoking phenotypes by using IVW method under two instrumental variable selection thresholds (1e-6 v.s. 5e-8).

**Figure S1:** Overview of the analytical plan and main findings.

To avoid overwhelming the reader, we only presented one of the key findings from our research as a representative example (pair of *Actinobacteria[p]*--Cigarettes Per Day)

**Figure S2**: Scatter plot of associations between genetic variants and *Actinobacteria[p]* versus between genetic variants and Cigarettes Per Day. The slope of each line represents the causal effect estimate using the corresponding MR analysis model, and the intercept can be interpreted as an estimate of the average horizontal pleiotropic effect across the genetic variants.

**Figure S3**: Forest plot of individual SNP estimates and summary estimates for the causal associations between *Actinobacteria[p]* abundance and Cigarettes Per Day.

**Figure S4**: Leave-one-out plot to assess if a single SNP drives the causal association between *Actinobacteria[p]* abundance and Cigarettes Per Day.

**Figure S5**: Funnel plot of MR estimation for the causal association between *Actinobacteria[p]* abundance and Cigarettes Per Day.

**Table S1.** Self-inspection results of STROBE-MR checklist of recommended items to address in reports of Mendelian randomization studies^1^ ^2^

| **Item No.** | **Section** | **Checklist item** | **Page No.** | **Relevant text from manuscript** |
| --- | --- | --- | --- | --- |
| 1 | **TITLE and ABSTRACT** | Indicate Mendelian randomization (MR) as the study’s design in the title and/or the abstract if that is a main purpose of the study | 1 | Cross-talks Between Gut Microbiota and Tobacco Smoking: A Two-Sample Mendelian Randomization Study |
|  | **INTRODUCTION** |  |  |  |
| 2 | **Background** | Explain the scientific background and rationale for the reported study. What is the exposure? Is a potential causal relationship between exposure and outcome plausible? Justify why MR is a helpful method to address the study question | 4-6 | Indeed, mounting observational evidence has reported that tobacco use was associated with alterations in gut microbiota composition.  Given the essential role of gut microbiota in the regulation of the central nervous system (CNS), another interesting question is whether smoking behaviors are affected by gut microbiota?  Associations between microbiome and smoking could be bridged by relevant metabolites.  The main reason for its advantage in inferring causality is that MR employs the genetic variants as instrumental variables. |
| 3 | **Objectives** | State specific objectives clearly, including pre-specified causal hypotheses (if any). State that MR is a method that, under specific assumptions, intends to estimate causal effects | 6-7 | Based on knowledge above, we hypothesized that gut microbiome links smoking behaviors and conducted a two-sample bi-directional MR analysis to elucidate the causal association between gut microbiota and smoking phenotypes, and further explore the potential role of several metabolites on these associations.  MR uses the facts that (1) genetic variants are randomly inherit one allele from each of the father and mother (namely the law of segregation assortment) and (2) alleles will be passed to offspring independently of each other (namely the law of independent assortment). Therefore, MR results are unlikely to be influenced by the environment that might confound the estimated relationship. |
|  | **METHODS** |  |  |  |
| 4 | **Study design and data sources** | Present key elements of the study design early in the article. Consider including a table listing sources of data for all phases of the study. For each data source contributing to the analysis, describe the following: |  |  |
|  | a) | Setting: Describe the study design and the underlying population, if possible. Describe the setting, locations, and relevant dates, including periods of recruitment, exposure, follow-up, and data collection, when available. | 7 and Table 1 | Detailed information, such as recruitment criteria of population and quality control of genetic data, can be found in the original paper (Table 1). |
|  | b) | Participants: Give the eligibility criteria, and the sources and methods of selection of participants. Report the sample size, and whether any power or sample size calculations were carried out prior to the main analysis | 7 and Table 1 | Detailed information, such as recruitment criteria of population and quality control of genetic data, can be found in the original paper (Table 1). |
|  | c) | Describe measurement, quality control and selection of genetic variants | 7-9 and Table 1 | Detailed information, such as recruitment criteria of population and quality control of genetic data, can be found in the original paper (Table 1). |
|  | d) | For each exposure, outcome, and other relevant variables, describe methods of assessment and diagnostic criteria for diseases | 7 and Table 1 | Detailed information, such as recruitment criteria of population and quality control of genetic data, can be found in the original paper (Table 1). |
|  | e) | Provide details of ethics committee approval and participant informed consent, if relevant | 7 | The data analyzed in this secondary study is publicly available from existing, published GWASs and therefore the ethical approval and informed consent have been obtained by all original studies. |
| 5 | **Assumptions** | Explicitly state the three core IV assumptions for the main analysis (relevance, independence and exclusion restriction) as well assumptions for any additional or sensitivity analysis | 9, 10 | The selection of IVs, the key to ensure the accuracy and robustness of the causal inferences, must meet MR's three key assumptions (Figure 1).  Three key assumptions of MR: (1) genetic variants must be associated with exposures; (2) genetic variants must not be associated with confounders; (3) genetic variants must affect outcomes only through exposures, not through other pathways. |
| 6 | **Statistical methods: main analysis** | Describe statistical methods and statistics used |  |  |
|  | a) | Describe how quantitative variables were handled in the analyses (i.e., scale, units, model) | Table 1 | Description column  For example, 1-SD increase in the lifetime smoking index was scaled to an individual smoking 20 cigarettes a day for 15 years and quitting 17 years ago, or smoking 60 cigarettes a day for 13 years and quitting 22 years ago. |
|  | b) | Describe how genetic variants were handled in the analyses and, if applicable, how their weights were selected | 9 | Firstly, palindromic variants with minor allele frequency greater than 0.4 were excluded; secondly, variants and their alleles were harmonized between the GWAS results of exposure and outcome; thirdly, independent SNPs (LD r2 < 0.01 and clumping distance = 250 kb, based on the European-based 1,000 Genome Projects reference panel) were selected at a compromised significant level. |
|  | c) | Describe the MR estimator (e.g. two-stage least squares, Wald ratio) and related statistics. Detail the included covariates and, in case of two-sample MR, whether the same covariate set was used for adjustment in the two samples | 10 | The list of covariates varies between original GWASs, but always included sex and age. Details can be found in the original studies. |
|  | d) | Explain how missing data were addressed | 7 | Detailed information, such as recruitment criteria of population and quality control of genetic data, can be found in the original paper (Table 1). |
|  | e) | If applicable, indicate how multiple testing was addressed | 11-12 | The statistical significance of the MR effect estimates was defined as a false discovery rate (FDR) of <10%, where the Benjamini-Hochberg procedure was used to correct for the number of taxa tested, accounting for multiple comparisons. |
| 7 | **Assessment of assumptions** | Describe any methods or prior knowledge used to assess the assumptions or justify their validity | 11,  Figure 2, and Figure 3 | Different approaches yield valid evidence under different assumptions. cML-MA, which without relying on the InSIDE assumption, was ......Parameter setting was the same as for univariate MR.  Evidence from functional studies were reported and displayed in the figures. |
| 8 | **Sensitivity analyses and additional analyses** | Describe any sensitivity analyses or additional analyses performed (e.g. comparison of effect estimates from different approaches, independent replication, bias analytic techniques, validation of instruments, simulations) | 11 | Different approaches yield valid evidence under different assumptions. cML-MA, which without relying on the InSIDE assumption, was ......Parameter setting was the same as for univariate MR. |
| 9 | **Software and pre-registration** |  |  |  |
|  | a) | Name statistical software and package(s), including version and settings used | 11 | MR were performed using the “TwoSampleMR” (version 0.5.6), “MRcML” (version 0.0.0.9), and “MendelianRandomization” (version 0.6.0) packages in R (version 4.1.2). |
|  | b) | State whether the study protocol and details were pre-registered (as well as when and where) | No Applicable | This is a secondary analysis based on summary statistics from existing, published studies. The ethical approval and informed consent have been obtained by all original studies. |
|  | **RESULTS** |  |  |  |
| 10 | **Descriptive data** |  |  |  |
|  | a) | Report the numbers of individuals at each stage of included studies and reasons for exclusion. Consider use of a flow diagram | 7 | Detailed information, such as recruitment criteria of population and quality control of genetic data, can be found in the original paper (Table 1). |
|  | b) | Report summary statistics for phenotypic exposure(s), outcome(s), and other relevant variables (e.g. means, SDs, proportions) | 7-9 | The genetic instrument variables (IVs), typically single-nucleotide polymorphisms (SNPs), for gut microbiota were retrieved from a large-scale GWAS meta-analysis, which contained 18,340 European-dominated participants from 24 separate cohorts with 5,717,754 SNPs after imputation......We extracted genetic data for these specific human blood metabolites (i.e., tryptophan, tyrosine, phenylalanine, glutamate, glycine, and valerate) from a GWAS comprising 7,824 European adult individuals.  Details can be found in the original studies. |
|  | c) | If the data sources include meta-analyses of previous studies, provide the assessments of heterogeneity across these studies | 7 | Detailed information, such as recruitment criteria of population and quality control of genetic data, can be found in the original paper (Table 1). |
|  | d) | For two-sample MR:  i.  Provide justification of the similarity of the genetic variant-exposure associations between the exposure and outcome samples  ii.  Provide information on the number of individuals who overlap between the exposure and outcome studies | 7 | These GWAS sample populations needed to be predominantly of European descent and largely independent of each other |
| 11 | **Main results** |  |  |  |
|  | a) | Report the associations between genetic variant and exposure, and between genetic variant and outcome, preferably on an interpretable scale | Table 1 | Description column  For example, 1-SD increase in the lifetime smoking index was scaled to an individual smoking 20 cigarettes a day for 15 years and quitting 17 years ago, or smoking 60 cigarettes a day for 13 years and quitting 22 years ago. |
|  | b) | Report MR estimates of the relationship between exposure and outcome, and the measures of uncertainty from the MR analysis, on an interpretable scale, such as odds ratio or relative risk per SD difference | 12-14, 23 | The results of IVW analyses showed that the genetic liability for smoking initiation had a causal contribution to an increased abundance of Intestinimonas[g] (Beta ± SE: 0.265 ± 0.090, P = 3.15e-03).  The limited sample size may also prevent us from providing a sufficiently precise estimate as well as 95% confidence intervals for clinical practice. |
|  | c) | If relevant, consider translating estimates of relative risk into absolute risk for a meaningful time period | No Applicable |  |
|  | d) | Consider plots to visualize results (e.g. forest plot, scatterplot of associations between genetic variants and outcome versus between genetic variants and exposure) | 14 | Additional visualizations of the results, including scatter plot, forest plot, leave-one-out plot, and leave-one-out plot can be found in Additional file 1: Figure S1-S4. |
| 12 | **Assessment of assumptions** |  |  |  |
|  | a) | Report the assessment of the validity of the assumptions | 13, | Neither horizontal pleiotropy nor heterogeneity (among IVs) was detected at statistically significant levels (all P _for Egger intercept_ > 0.05, most of the P _for PRESSSO global test_ > 0.05, and all P f_or Cochran’s Q_ > 0.05). |
|  | b) | Report any additional statistics (e.g., assessments of heterogeneity across genetic variants, such as *I^2^*, Q statistic or E-value) | 13 and Table S1 | Neither horizontal pleiotropy nor heterogeneity (among IVs) was detected at statistically significant levels (all P _for Egger intercept_ > 0.05, most of the P _for PRESSSO global test_ > 0.05, and all P f_or Cochran’s Q_ > 0.05). |
| 13 | **Sensitivity analyses and additional analyses** |  |  |  |
|  | a) | Report any sensitivity analyses to assess the robustness of the main results to violations of the assumptions | 13 and Table S1 | Neither horizontal pleiotropy nor heterogeneity (among IVs) was detected at statistically significant levels (all P _for Egger intercept_ > 0.05, most of the P _for PRESSSO global test_ > 0.05, and all P f_or Cochran’s Q_ > 0.05).  Additionally, the results of MVMR-Egger indicated that our multivariable MR estimates were unlikely biased by pleiotropy (most of P for MVMR-Egger intercept > 0.05). |
|  | b) | Report results from other sensitivity analyses or additional analyses | 14 | Moreover, the MR results evaluated under two instrumental variable selection thresholds (1e-6 v.s. 5e-8) were presented in Additional file 1: Table S3, which indicated very limited difference on β-coefficients (r _microbiota to smoking_ = 0.99, P < 0.001). |
|  | c) | Report any assessment of direction of causal relationship (e.g., bidirectional MR) | 12-15 | Causal effect of smoking on gut microbiota  Causal effect of gut microbiota on smoking |
|  | d) | When relevant, report and compare with estimates from non-MR analyses | 12-15, Figure 2, and Figure 3 | The results of IVW analyses showed that the genetic liability for smoking initiation had a causal contribution to an increased abundance of Intestinimonas[g] (Beta ± SE: 0.265 ± 0.090, P = 3.15e-03), which was in line with the evidence from a mice model showing that exposure to the major cigarette smoke carcinogens (NNK plus BaP) could elevate fecal level of Intestinimonas (Figure 2a).  Evidence from functional studies were reported and displayed in the figures. |
|  | e) | Consider additional plots to visualize results (e.g., leave-one-out analyses) | 14 | Additional visualizations of the results, including scatter plot, forest plot, and leave-one-out plot can be found in Additional file 1: Figure S1-S4. |
|  | **DISCUSSION** |  |  |  |
| 14 | **Key results** | Summarize key results with reference to study objectives | 17 | This two-sample MR study gave reasonably strong evidence that genetically predicted abundance of specific gut microbes play non-negligible roles in the occurrence and progression of cigarette smoking, in which, metabolites may be participating. As for the other direction, the MR confirmed and strengthened the role of smoking on gut microbiota. |
| 15 | **Limitations** | Discuss limitations of the study, taking into account the validity of the IV assumptions, other sources of potential bias, and imprecision. Discuss both direction and magnitude of any potential bias and any efforts to address them | 22 | Several limitations of our study should be acknowledged. Firstly...... |
| 16 | **Interpretation** |  |  |  |
|  | a) | Meaning: Give a cautious overall interpretation of results in the context of their limitations and in comparison with other studies | 18-19, 20-22 | Leveraging the large-scale GWAS data sources, our MR study filled this knowledge gap from a novel angle. (1)...(2)...  Rather than just concerning the causality between gut microbiota and smoking, we also considered the possible involvement of metabolites in this process. (1)...(2)...  In the other direction, our findings strengthened and extended existing observational evidence, suggesting that tobacco smoking could disrupt the homeostasis of the intestinal microbiota. (1)...(2)... |
|  | b) | Mechanism: Discuss underlying biological mechanisms that could drive a potential causal relationship between the investigated exposure and the outcome, and whether the gene-environment equivalence assumption is reasonable. Use causal language carefully, clarifying that IV estimates may provide causal effects only under certain assumptions | 19-20, 22 | There is growing evidence, albeit some indirect, providing possible biological explanations for the mechanisms of commensal gut microbiota on smoking, particularly probiotics such as Bifidobacterium. (1)...(2)...(3)...  The main mechanisms by which smoking affects the gut microbiota include the following: raising the pH of the intestinal environment, inducing chronic low-grade inflammation or inflammation-related diseases, as well as promoting oxidative stress. |
|  | c) | Clinical relevance: Discuss whether the results have clinical or public policy relevance, and to what extent they inform effect sizes of possible interventions | 23, 23-24 | Likewise, the estimates of a lifetime effect of gut microbiota on smoking provided by MR cannot deliver much clinical meaningful for age-specific interventions. The limited sample size may also prevent us from providing a sufficiently precise estimate as well as 95% confidence intervals for clinical practice.  Taking together the existing evidence, potential mechanisms including a positive feedback loop of smoking and the potential role of neurotransmitter-associated metabolic biomarkers therein were revealed. Our study highlighted the hazards of tobacco use for gut flora dysbiosis and shed light on the potential role of specific gut microbiota for tobacco use behaviors. It would be helpful to perform a gender- or age-specific MR analysis especially with larger sample size in future endeavors. |
| 17 | **Generalizability** | Discuss the generalizability of the study results (a) to other populations, (b) across other exposure periods/timings, and (c) across other levels of exposure | 22, 23 | Thirdly, although most of the participants of the gut microbial GWAS were ancestrally Europeans, the ethnic proportion was not perfectly matched between the two samples (i.e., the exposure GWAS and the outcome GWAS dataset), which may result in some levels of inconsistency in LD correlations.  Likewise, the estimates of a lifetime effect of gut microbiota on smoking provided by MR cannot deliver much clinical meaningful for age-specific interventions. The limited sample size may also prevent us from providing a sufficiently precise estimate as well as 95% confidence intervals for clinical practice. It would be helpful to perform a gender- or age-specific MR analysis especially with larger sample size in future endeavors. |
|  | **OTHER INFORMATION** |  |  |  |
| 18 | **Funding** | Describe sources of funding and the role of funders in the present study and, if applicable, sources of funding for the databases and original study or studies on which the present study is based | 25 | Funding Sources |
| 19 | **Data and data sharing** | Provide the data used to perform all analyses or report where and how the data can be accessed, and reference these sources in the article. Provide the statistical code needed to reproduce the results in the article, or report whether the code is publicly accessible and if so, where | 25 | Availability of data and materials |
| 20 | **Conflicts of Interest** | All authors should declare all potential conflicts of interest | 26 | The authors declare that they have no conflict of interest. |

This checklist is copyrighted by the Equator Network under the Creative Commons Attribution 3.0 Unported (CC BY 3.0) license.

1. Skrivankova VW, Richmond RC, Woolf BAR, Yarmolinsky J, Davies NM, Swanson SA, et al. Strengthening the Reporting of Observational Studies in Epidemiology using Mendelian Randomization (STROBE-MR) Statement. JAMA. 2021;under review.

2. Skrivankova VW, Richmond RC, Woolf BAR, Davies NM, Swanson SA, VanderWeele TJ, et al. Strengthening the Reporting of Observational Studies in Epidemiology using Mendelian Randomisation (STROBE-MR): Explanation and Elaboration. BMJ. 2021;375:n2233.

**Table S2.** Self-inspection results of critical appraisal checklist proposed by Davies et al. for evaluating Mendelian randomization studies ^[1]^

| **Item NO.** | **Section** | **Checklist item** | **Page No.** |
| --- | --- | --- | --- |
| 1. | Core MR assumptions | Is there sufficient evidence that the genetic variants are robustly associated with the risk factor of interest? | 9 |
| 2 |  | Are the genetic variants associated with potential confounders? Do the authors present this relationship? | 10 |
| 3 |  | Is there any way for the genetic variants to affect the outcome through alternative pathways (horizontal pleiotropy)? Do the authors present alternative Mendelian randomisation approaches (such as MR Egger, median, and mode estimators, or use of “negative control” populations) to investigate this more fully? | 10-11 |
| 4 | Methods reporting | Are the effect and other alleles coded in the same direction for the exposure and outcome? | 9 |
| 5 |  | Were the two samples drawn from the same population? | 7 |
| 6 |  | Were the two samples independent? | 7 |
| 7 |  | Was the analysis restricted to independent variants (that is, pruned of SNPs in linkage disequilibrium) or did the analysis allow for the correlation between variants? | 9 |
| 8 | Data presentation | Do the authors present the results as a genetic association, an instrumental variable estimate, or both? | 12-15 |
| 9 |  | If they provide an instrumental variable estimate, do they compare it with the conventional observational estimate? | 18-19 |
| 10 |  | Do the authors provide sensitivity analyses such as MR Egger, weighted median, and mode Mendelian randomisation, or use negative control populations? | 13, 14, 16 |
| 11 |  | Do the authors manually pick and choose which SNPs go into the instrument to tackle pleiotropy? If so, is the approach and justification clear? | No Applicable  Pleiotropy was estimated using MR-Egger, WME, MR-PRESSO, and cML-MA. No manual IV selections. |
| 12 |  | Do the authors provide the data that they used (especially for Mendelian randomisation analyses conducted at the summary level) in a supplement to allow researchers to reproduce their findings? | Table 1 |
| 13 | Interpretation | If the Mendelian randomisation estimate is similar to the observational estimate and provides evidence in support of a causal effect, could it be due to weak instrument bias in a single study or confounding through, for example, horizontal pleiotropy? | 18-19 |
| 14 |  | If the Mendelian randomisation estimate differs from the observational estimate and provides little evidence of a causal effect, could this be due to weak instrument bias when using two different samples or negative confounding due to pleiotropy? | 18-19 |
| 15 |  | Mendelian randomisation provides estimates of the effects of the risk factor over a lifetime, and the numerical effect estimates may not be clinically meaningful. Will interventions at a specific age have the same sized effects? | 23 |
| 16 |  | Are the 95% confidence intervals of the Mendelian randomisation estimate sufficiently precise to identify the observational estimate and a clinically meaningful difference? | 23 |
| 17 | Clinical implications | Do the results triangulate with other forms of evidence? Could a clinical trial be conducted to provide definitive evidence, as in the case of PCSK9 inhibitors? If a randomised clinical trial is not feasible (such as in the case of alcohol consumption and risk of heart disease) or unlikely to be conducted in the short term (such as the case of lifestyle interventions to lower BMI and risk of heart disease), and there is existing evidence from multiple Mendelian randomisation studies and other robust study designs that converge on a similar result and show consistency of association, this information can be used to guide patient care; for example, advising weight loss to prevent heart disease or advising against moderate alcohol consumption to prevent cardiovascular disease | 23 |

[1] Davies NM, Holmes MV, Davey Smith G. Reading Mendelian randomisation studies: a guide, glossary, and checklist for clinicians. BMJ. 2018 Jul 12;362:k601. doi: 10.1136/bmj.k601.

| **Table S3** The MR results of causal links between gut microbiome and smoking phenotypes by using IVW method  under two instrumental variable selection thresholds (1e-6 v.s. 5e-8). | | | | | | |
| --- | --- | --- | --- | --- | --- | --- |
| **Exposure** | **Outcome** | **No.SNP** | **Threshold** | ***β*** | **SE** | ***P*_IVW_** |
| phylum.*Actinobacteria* | Age Of Initiation | 6 | 1e-6 | 0.051 | 0.019 | 7.74e-03 |
|  |  | 4 | 5e-8 | 0.057 | 0.022 | 0.011 |
| order.*Bifidobacteriales* |  | 7 | 1e-6 | 0.050 | 0.016 | 1.73e-03 |
|  |  | 6 | 5e-8 | 0.047 | 0.018 | 7.71e-03 |
| family.*Bifidobacteriaceae* |  | 7 | 1e-6 | 0.050 | 0.016 | 1.73e-03 |
|  |  | 6 | 5e-8 | 0.047 | 0.018 | 7.71e-03 |
| genus.*Bifidobacterium* |  | 7 | 1e-6 | 0.049 | 0.016 | 1.79e-03 |
|  |  | 6 | 5e-8 | 0.046 | 0.017 | 7.58e-03 |
|  | | | | | | |
| phylum.*Actinobacteria* | Cigarettes Per Day | 6 | 1e-6 | -0.066 | 0.024 | 5.31e-03 |
|  |  | 4 | 5e-8 | -0.079 | 0.032 | 0.014 |
| class.*Actinobacteria* |  | 8 | 1e-6 | -0.053 | 0.018 | 3.70e-03 |
|  |  | 5 | 5e-8 | 0.063 | 0.022 | 4.25e-03 |
| order.*Bifidobacteriales* |  | 7 | 1e-6 | -0.048 | 0.019 | 1.12e-02 |
|  |  | 6 | 5e-8 | -0.051 | 0.021 | 0.014 |
| family.*Bifidobacteriaceae* |  | 7 | 1e-6 | -0.048 | 0.019 | 1.12e-02 |
|  |  | 6 | 5e-8 | -0.051 | 0.021 | 0.014 |
| genus.*Bifidobacterium* |  | 7 | 1e-6 | -0.048 | 0.018 | 8.83e-03 |
|  |  | 6 | 5e-8 | -0.051 | 0.020 | 0.011 |
|  | | | | | | |
| phylum.*Actinobacteria* | Lifetime Smoking | 6 | 1e-6 | -0.023 | 0.009 | 8.43e-03 |
|  |  | 4 | 5e-8 | -0.022 | 0.010 | 0.027 |
| class.*Actinobacteria* |  | 7 | 1e-6 | -0.019 | 0.007 | 5.79e-03 |
|  |  | 5 | 5e-8 | -0.020 | 0.007 | 5.47e-03 |
| order.*Bifidobacteriales* |  | 6 | 1e-6 | -0.023 | 0.008 | 5.32e-03 |
|  |  | 5 | 5e-8 | -0.019 | 0.007 | 6.17e-03 |
| family.*Bifidobacteriaceae* |  | 6 | 1e-6 | -0.023 | 0.008 | 5.32e-03 |
|  |  | 5 | 5e-8 | -0.019 | 0.007 | 6.17e-03 |
| genus.*Bifidobacterium* |  | 6 | 1e-6 | -0.023 | 0.008 | 7.00e-03 |
|  |  | 5 | 5e-8 | -0.019 | 0.007 | 6.63e-03 |
| genus.*Peptococcus* |  | 4 | 1e-6 | -0.019 | 0.007 | 7.30e-03 |
|  |  | <3 | 5e-8 | - | - | - |
|  | | | | | | |
| Age Of Initiation | genus.*Eisenbergiella* | 24 | 1e-6 | -1.062 | 0.250 | 2.21e-05 |
|  |  | 8 | 5e-8 | -0.805 | 0.451 | 0.074 |
|  | genus.*Lactococcus* | 23 | 1e-6 | 1.135 | 0.317 | 3.45e-04 |
|  |  | 8 | 5e-8 | 1.079 | 0.421 | 0.010 |
|  | | | | | | |
| Smoking Initiation | order.*Pasteurellales* | 287 | 1e-6 | -0.328 | 0.097 | 7.40e-04 |
|  |  | 128 | 5e-8 | -0.302 | 0.138 | 0.029 |
|  | family.*Pasteurellaceae* | 287 | 1e-6 | -0.328 | 0.097 | 7.40e-04 |
|  |  | 128 | 5e-8 | -0.302 | 0.138 | 0.029 |
|  | family.*Christensenellaceae* | 291 | 1e-6 | -0.278 | 0.072 | 1.18e-04 |
|  |  | 130 | 5e-8 | -0.302 | 0.097 | 1.96e-03 |
|  | genus.*ChristensenellaceaeR* | 291 | 1e-6 | -0.267 | 0.073 | 2.75e-04 |
|  |  | 130 | 5e-8 | -0.295 | 0.099 | 2.86e-03 |
|  | genus.*Haemophilus* | 287 | 1e-6 | -0.321 | 0.099 | 1.15e-03 |
|  |  | 128 | 5e-8 | -0.283 | 0.141 | 0.045 |
|  | genus.*Intestinimonas* | 290 | 1e-6 | 0.265 | 0.090 | 3.15e-03 |
|  |  | 129 | 5e-8 | 0.351 | 0.120 | 3.55e-03 |
|  | genus.*Romboutsia* | 290 | 1e-6 | -0.279 | 0.085 | 1.06e-03 |
|  |  | 129 | 5e-8 | -0.285 | 0.110 | 9.94e-03 |
|  | | | | | | |
| Lifetime Smoking | class.*Coriobacteriia* | 372 | 1e-6 | 0.242 | 0.069 | 4.72e-04 |
|  |  | 185 | 5e-8 | 0.234 | 0.088 | 8.25e-03 |
|  | order.*Coriobacteriales* | 372 | 1e-6 | 0.242 | 0.069 | 4.72e-04 |
|  |  | 185 | 5e-8 | 0.234 | 0.088 | 8.25e-03 |
|  | family.*Coriobacteriaceae* | 372 | 1e-6 | 0.242 | 0.069 | 4.72e-04 |
|  |  | 185 | 5e-8 | 0.234 | 0.088 | 8.25e-03 |
|  | genus.*Catenibacterium* | 328 | 1e-6 | 0.505 | 0.170 | 2.98e-03 |
|  |  | 162 | 5e-8 | 0.563 | 0.236 | 0.017 |
|  | genus.*RuminococcaceaeNK4A214* | 371 | 1e-6 | -0.261 | 0.074 | 4.48e-04 |
|  |  | 185 | 5e-8 | -0.255 | 0.098 | 9.48e-03 |
|  | genus.*RuminococcaceaeUCG005* | 371 | 1e-6 | -0.237 | 0.076 | 1.91e-03 |
|  |  | 185 | 5e-8 | -0.233 | 0.107 | 0.029 |
|  | genus.*Eubacterium xylanophilum* | 370 | 1e-6 | -0.308 | 0.080 | 1.08e-04 |
|  |  | 184 | 5e-8 | -0.387 | 0.104 | 1.84e-04 |
| IVW, inverse-variance weighted; No.SNP, number of single-nucleotide polymorphism (SNP); SE, standard error. | | | | | | |

**Figure S1:** Overview of the analytical plan and main findings.


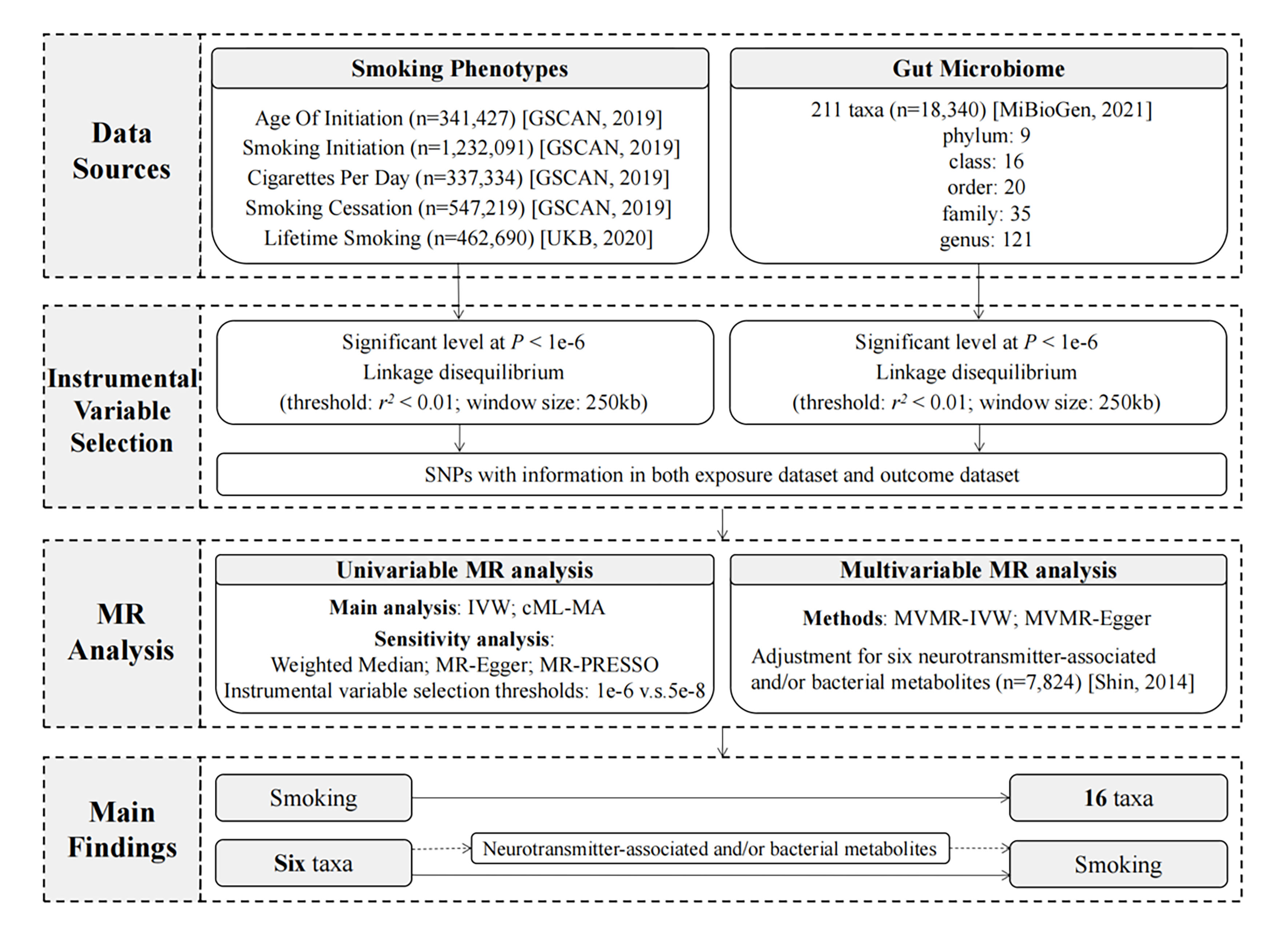


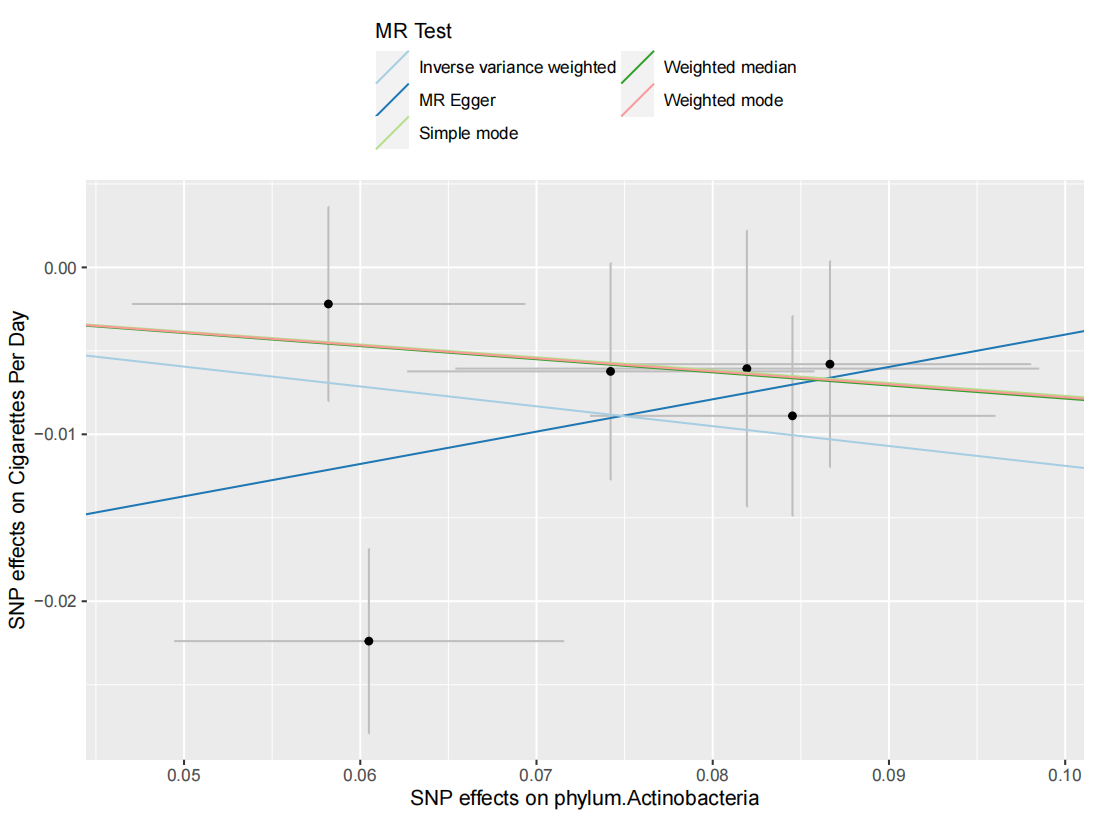
**Figure S2:** Scatterplot of associations between genetic variants and *Actinobacteria[p]* versus between genetic variants and Cigarettes Per Day. The slope of each line represents the causal effect estimate using the corresponding MR analysis model, and the intercept can be interpreted as an estimate of the average horizontal pleiotropic effect across the genetic variants.

**Figure S3:** Forest plots of individual SNP estimates and summary estimates for the causal associations between *Actinobacteria[p]* abundance and Cigarettes Per Day.

**
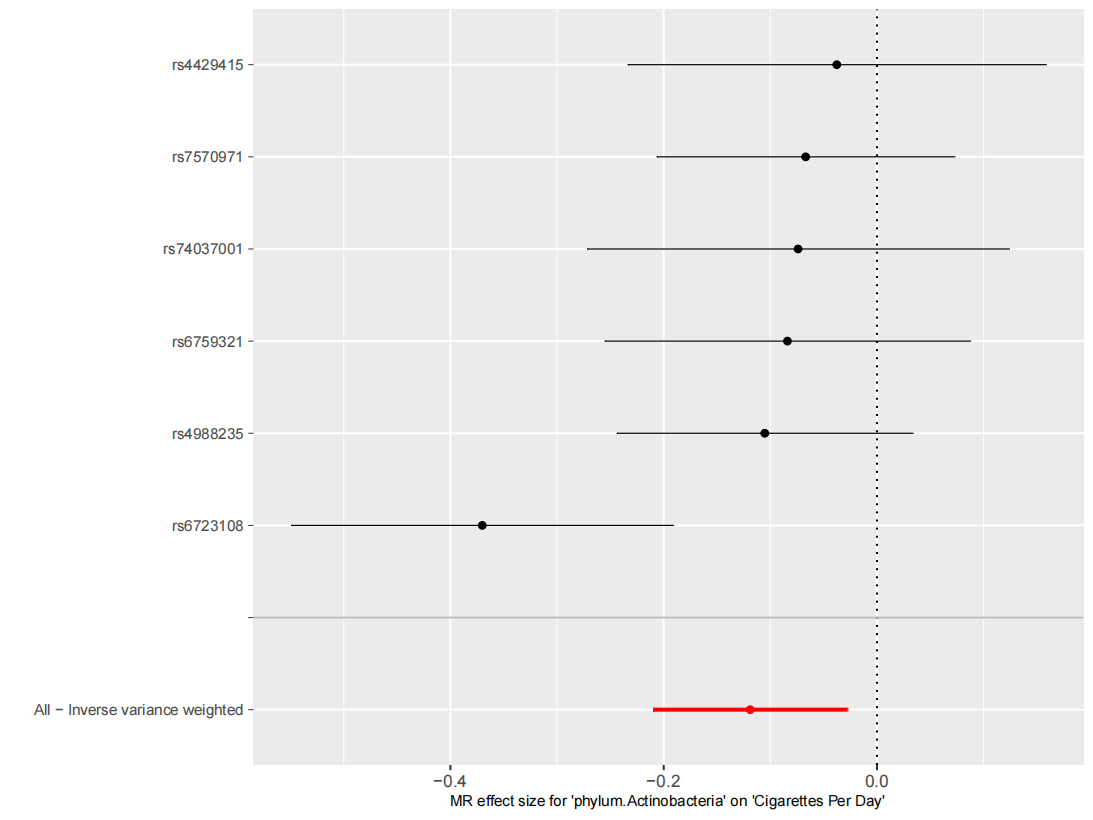
Figure S4:** Leave-one-out plot to assess if a single SNP drives the causal association between *Actinobacteria[p]* abundance and Cigarettes Per Day.


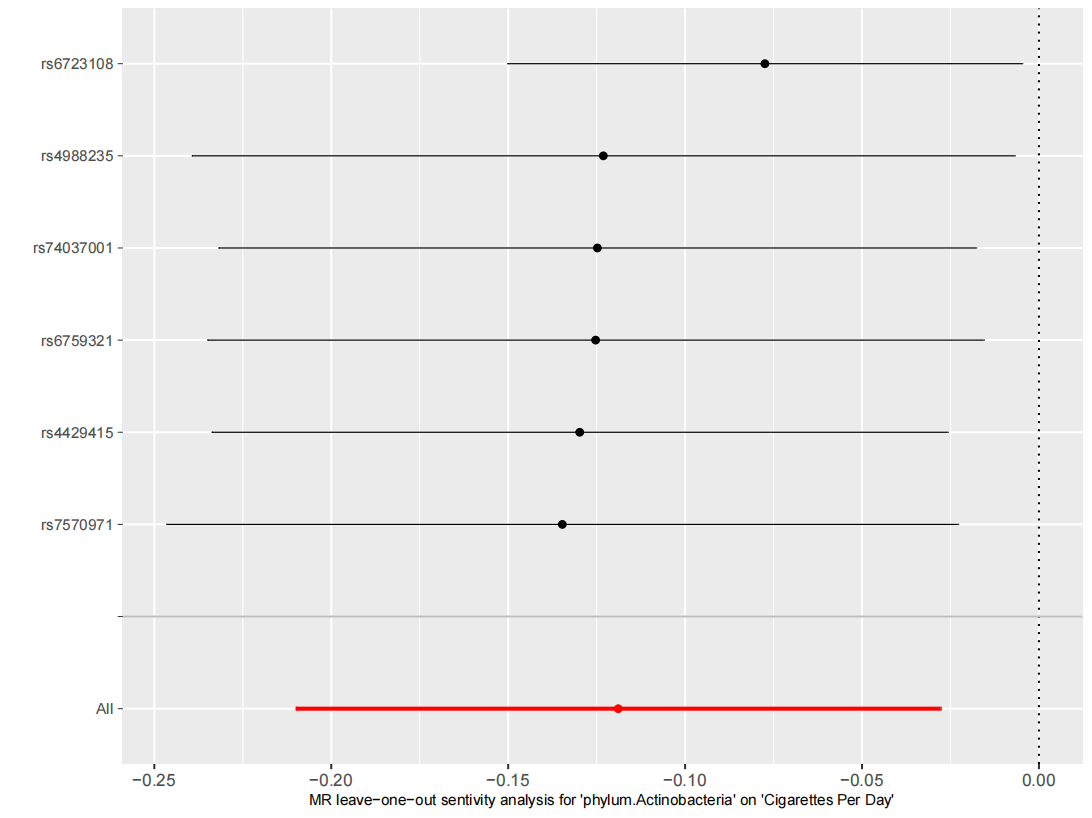


**Figure S5:** Funnel plots of MR estimation for the causal association between *Actinobacteria[p]* abundance and Cigarettes Per Day.


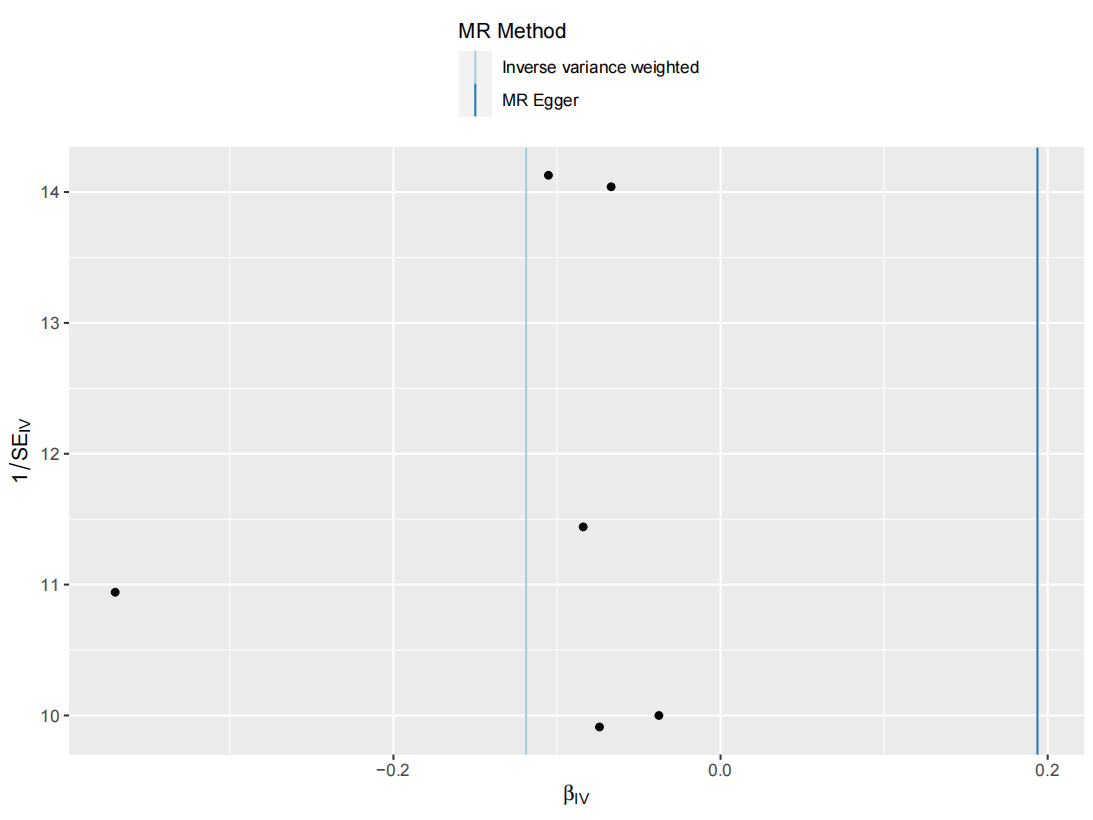

Supplement: Supplementary file 1 — Additional file 1: Table S1. Self-inspection results of STROBE-MR checklist of recommended items to address in reports of Mendelian randomization studies. Table S2. Self-inspection results of critical appraisal checklist proposed by Davies et al. for evaluating Mendelian randomization studies. Table S3. The MR results of causal links between gut microbiome and smoking phenotypes by using IVW method under two instrumental variable selection thresholds. Figure S1. Overview of the analytical plan and main findings. Figure S2. Scatter plot of associations between genetic variants and Actinobacteria[p] versus between genetic variants and Cigarettes Per Day. The slope of each line represents the causal effect estimate using the corresponding MR analysis model, and the intercept can be interpreted as an estimate of the average horizontal pleiotropic effect across the genetic variants. Figure S3. Forest plot of individual SNP estimates and summary estimates for the causal associations between Actinobacteria[p] abundance and Cigarettes Per Day. Figure S4. Leave-one-out plot to assess if a single SNP drives the causal association between Actinobacteria[p] abundance and Cigarettes Per Day. Figure S5. Funnel plot of MR estimation for the causal association between Actinobacteria[p] abundance and Cigarettes Per Day. [file 12916_2023_2863_MOESM1_ESM.docx]
